# A review of public opinion towards alcohol controls in Australia

**DOI:** 10.1186/1471-2458-11-58

**Published:** 2011-01-27

**Authors:** Claire Tobin, A Rob Moodie, Charles Livingstone

**Affiliations:** 1 Department of Health and Social Science, School of Public Health and Preventive Medicine, Monash University, PO Box 197 Caulfield East, Victoria 3145 Australia; 2 Nossal Institute for Global Health, Faculty of Medicine, Dentistry and Health Sciences, University of Melbourne, Carlton, Victoria 3010 Australia

## Abstract

**Background:**

Increasing concern about the negative impact of alcohol on the Australian community has renewed calls for tighter regulatory controls. This paper reviews levels of and trends in public support for liquor control regulations, regulation of alcohol promotions, and alcohol pricing and taxation reforms in Australia between 1998 and 2009.

**Methods:**

Six electronic databases and twenty public health and alcohol organisation websites were searched for research literature, reports and media releases describing levels of public support for alcohol controls. Only studies which randomly selected participants were included.

**Results:**

Twenty-one studies were included in the review. The majority of the Australian public support most proposed alcohol controls. Levels of support are divided between targeted and universal controls.

**Conclusions:**

Implementation of targeted alcohol policies is likely to be strongly supported by the Australian public, but universal controls are liable to be unpopular. Policy makers are provided with insights into factors likely to be associated with higher public support.

## Background

Australia begins a new decade with concerns about its drinking culture and alcohol-related harm high on the public health agenda. A relentless media focus on a "drunken" drinking culture and alcohol-related violence in inner cities [1-3], increased demands on law enforcement and health services to respond to alcohol-related incidents [4,5], and widespread public concern about the effects of alcohol on the community [6] are intensifying the pressure on all levels of government to take action.

Consumption of alcohol in Australia is high by world standards [7]; 83% of Australians aged 14 years and over consume alcohol, one-in-five regularly drink at levels which risk short-term harm, and one-in-ten at levels which risk long-term harm [8]. In addition to increased personal risk of morbidity and mortality, alcohol-related harm to third parties have become so common that the term "passive drinking" has been coined to denote the impact of drunken behaviour on others [9-11]. Harmful alcohol consumption impacts on public safety, family violence, workplace productivity, household functioning and road accidents at an estimated annual cost to the Australian community of $15 billion [10,12].

In June 2009 the National Preventative Health Taskforce presented the Federal Minister for Health and Ageing with a comprehensive package of recommendations across several key policy areas to reshape the drinking culture in Australia and reduce the harm from alcohol, so that Australia can begin the next decade as the "healthiest country" [13].

The Government responded to the Taskforce's recommendations eleven months later [14], revealing reluctance to adopt recommended regulatory action in areas under the Federal remit such as taxation and promotion. This cautious approach is perhaps understandable for a democratic government nearing the end of its first term. Although 80% of Australians acknowledge that Australia has a national drinking problem [15], it does not necessarily follow that the public will support increased regulation of a popular, regularly consumed commodity which appears integral to the national culture.

A dynamic relationship exists between public opinion and policy; public opinion more often than not has an influence on policy, and policy can shape public opinion [16]. The strength of this relationship is mediated by both the salience of the issue and the presence and power of other interested parties [17]. Given an issue as prominent as alcohol, and with many vested interests, there is no doubt that policy makers pay close attention to Australians' opinions on alcohol control policy.

Numerous studies have documented public opinion towards alcohol policies in Australia, Canada, the United States of America, and Europe in the past twenty years [18-22]. Similar trends in public opinion towards alcohol controls are apparent across these jurisdictions. In summary, public support for alcohol controls is divided. Support is lower for policies which seek to restrict the physical and economic availability of alcohol to the wider public, even though a strong evidence base establishes these controls as most effective for reducing alcohol consumption and alcohol-related harm [23,24], and higher for policies directed towards informing, educating and treating targeted individuals. Wilkinson *et al *(2009), analysing trends in Australian public opinion recorded by the National Drug Strategy Household Survey (NDSHS) between 1993 and 2004, found that support for alcohol controls gradually declined during this time. They also concluded that female and older aged Australians are more likely to support alcohol controls, and increasing volume and frequency of alcohol drinking is a strong predictor of opposition to alcohol controls [18].

Opinions on alcohol policy have been observed to reflect existing societal norms and drinking patterns [16]. Liberalised availability and increasing promotion of alcohol may have an impact on levels of public support for alcohol policy. In Ontario, a ten year period of dramatically increasing access to alcohol was accompanied by a parallel erosion of public support for alcohol controls [25]. Conversely, another study found that increasing awareness of alcohol-related harm in the community was associated with more supportive opinions of alcohol controls [22].

In Australia, a growing public awareness of tobacco-related harm, combined with strong coordinated advocacy and enhanced regulation was associated with increasing public support for tobacco control in the 1980-90s [26,27]. It is arguable that similar conditions now exist for alcohol, and that public support for alcohol control is following a similar pathway.

The aim of this review is to describe the Australian public's opinions towards regulatory controls to reduce the harm from alcohol. Specifically, the review draws on the latest available evidence to determine the proportion of Australians who support alcohol controls. Data from the last decade are utilised to test three hypotheses derived from previously observed trends in public opinion towards tobacco control:

1. public support for alcohol controls is increasing over time [28,29]

2. public support for alcohol controls increases following implementation of new regulation [30-32], and

3. public support is higher for alcohol controls which seek to protect children and innocent third parties from harm [27,31,33,34].

## Methods

### Search strategy

Six electronic databases (MEDLINE, INFORMIT, SCOPUS, WEB of SCIENCE, EBSCO, Google Scholar) were searched for research published between 1998 and 2009. Search terms were chosen to reflect the broad categories to which the research applies; "alcohol, drinking, binge-drinking" and "public, community, opinion, attitude, perception" and "regulation, control, legislation, tax, price, ban, restriction" and "Australia" (see Additional file 1 for example). Additional literature was sourced by searching citation managers and by reviewing reference lists. The last search was run on 23 February 2010.

Other publically available research publications, reports and opinion polls were sought by searching the websites of twenty key organisations pertinent to public health and alcohol and other related industries. Where reference to, or only part of the research was publicly accessible, the organisation which was custodian of the data was contacted to request a release of the full findings; where this was granted the research was included; where not, excluded.

### Study selection

**Figure 1:**
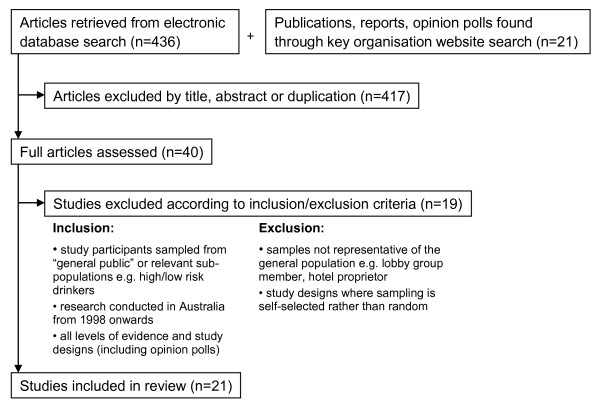
**Study selection flow chart**.

Literature was selected for initial inclusion by examining title and abstract for relevance to the aims of the review, and then more thoroughly assessed against set criteria for inclusion and exclusion (see Figure 1).

Mindful that nearly all public opinion data are collected from descriptive studies at one point in time, the selection criteria included all types of study designs, notwithstanding the 'very low' quality rating assigned to evidence obtained from non-experimental studies using systematic review type grading systems [35]. To reduce bias, studies were selected or excluded according to an appraisal of their sampling strategy; only studies where participants were randomly selected for participation in research were included. In order for the findings to reflect relatively contemporary opinions, only research conducted from 1998 to 2009 was selected for review.

### Data extraction

For all included studies, data were extracted and tabulated regarding the control type, level of support, question wording, study design, sampling strategy, sample population and size, response rate, jurisdiction and year data collected, and data source.

In the original studies level of support was recorded on a variety of response scales; six studies used a five-point Likert scale with a middle "neither" response [6,36-40], a further nine studies used the same scale but also allowed a "don't know", "refused" or "other" response [41-52], one study used a four point scale [53], and one used a two-point scale but also allowed a "don't know" or "unaware of the restriction" response [54]. The response scale of the remaining included studies was not reported. During data extraction, level of support data were condensed into a binary response of support or do not support.

### Quality appraisal

**Figure 2:**
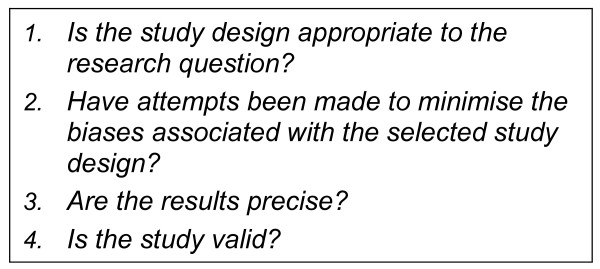
**Criteria for critical appraisal**.

The quality of each included study was critiqued using four key questions adapted from criteria specific to observational study designs [55] (see Figure 2). It was pre-determined that the quality assessment would be used only to distinguish between included studies, and not as a basis for exclusion.

Cross-sectional studies are the most convenient, least costly and commonly used study design for determining population prevalence outcomes such as public opinion [56], however their limitations include an inability to depict temporal relationships between exposure and outcomes, and vulnerability to selection bias [57,58]. Studies which attempted to minimise these limitations were deemed of higher quality and prioritised in the presentation of findings; studies which repeated measurements of public opinion over time were prioritised over single measurements, and studies with large sample sizes were prioritized over smaller samples because they were deemed more likely to reflect the variation in the true population. The size of the sample, geographic location of the sampling frame, and demographic similarity of the respondents were considered when assessing the ability of the results to be generalised to the true population. Acknowledging that the framing of questions affects the responses [59], the context and phrasing of the questions used by each study to elicit the measured opinions were examined closely when interpreting the findings.

### Data synthesis

The opinion data included in the review were measured using varied questioning-styles and sourced from non-homogenous populations sampled from different jurisdictions. It was therefore not appropriate to synthesise the data through a pooling of results using meta-analysis [60]. Instead, results were grouped by type of alcohol control and the range of findings over time was presented.

## Results

Twenty-one studies were included in the review [6,15,36-54,61-65]. Fifteen of the studies measure public opinion Australia-wide, and the remaining six were conducted in New South Wales (NSW), the Northern Territory (NT), Victoria, and Western Australia (WA). Data from three studies were sourced from peer review journals, eight from government and non-government reports, and ten from organisation websites or media releases.

Public support is described for three distinct key action areas in the National Preventative Health Strategy where further regulatory control of alcohol is recommended; liquor control regulations, regulation of alcohol promotions, and alcohol taxation and pricing reforms.

### Public support for liquor control regulations

Public support for a variety of liquor control regulations are presented in Additional file 2. Support is highest for initiatives which are directed towards licensees. A large nation-wide survey and a small NSW study conducted in 2007 found that more than four-fifths of Australians and three-quarters of rural NSW residents support stricter regulation, enforcement and penalties for irresponsible serving of alcohol [36,41,44].

Support is lower for liquor controls which seek to reduce the availability of alcohol. According to the 2007 NDSHS, 39% of Australians support reducing trading hours at pubs and clubs, and 33% support reducing alcohol outlet density [41]. Although less than majority support exists for these strategies, support in this policy area may be beginning to trend upwards [41-43,45].

Controls targeted towards high risk venues and populations are better supported than universal liquor controls. Public support for controls on late night venues and trading has increased between 2001 and 2007. The NDSHS found that 58% of Australians in 2007 would support restrictions on late night trading of alcohol (up from 51% in 2001) and 75% would support strict monitoring of late night premises (up from 60% in 2001) [41-43]. Higher support for restricted trading hours was recorded in Indigenous communities. A 2003 survey of Alice Springs town camp residents found that support for restricted alcohol trading hours at bars was 44% and for takeaways was 60% [54]. Community support for these additional restrictions increased during the 12 month period following initial implementation [54].

### Public support for regulation of alcohol promotions

Data presented in Additional file 3 demonstrates that public support for regulation of alcohol promotions is high. A 2009 opinion poll found that over two-thirds of the Australian public support government regulation of alcohol advertising and marketing [15], and two further studies indicate that there is high support for stronger restrictions on advertising than the status quo [48,61].

Support is slightly higher for controls which reduce young people's exposure to alcohol advertising - in three separate studies sampling Australia-wide and in Victoria between 2007 and 2009 approximately three-quarters of the public supported these measures [6,41,44,46]. Similar levels of support exist for reducing young people's exposure to television advertising which links alcohol and sport [62]. However endorsement for banning alcohol sponsorship of sport more broadly is lower; this is supported by 49% of Australians in 2007 and 45% of Western Australians in 2009 [41,44,47].

Very high support for including health warnings on alcohol containers and for requiring counter-advertising health warnings with all print media and television advertising of alcohol suggests the public recognise alcohol as a health problem. Over two-thirds of Australians in 2001 and 2006, and 89% of Victorians in 2009 agree that health warnings should be included on alcohol containers [49,63,64]. Seven out of ten Australians polled in 2006 supported counter-advertising [64].

### Public support for reforming alcohol taxation and pricing to discourage harmful drinking

The Australian public are least supportive of policy which seeks to control alcohol consumption using price and tax increases (see Additional file 4). Less than one-quarter of the Australian public in 2007 gave unqualified support for a price increase on alcohol [41]. However support by Australians at the same time was nearly doubled for an increased tax on alcohol [6,41,44] (which invariably would also increase price) and was as high as 67% in Victoria in 2009 [50], if the additional revenue collected is dedicated towards the prevention and treatment of alcohol-related harm.

Support is much higher for specific price and tax reforms in comparison to general price and tax increases. A large survey of Victorian residents in 2009 found that over two-thirds of respondents would support tiered tax rates according to increasing alcoholic content; 61% agreed that the highest tax rate should be applied to products that cause the greatest harm, and 79% supported a minimum price for bottled alcohol so that it is not cheaper than bottled water [50]. Higher public support for a minimum floor price and for volumetric and risk-based taxation on alcohol may be due to the more evident logical rationale for change which is grounded in these policies. Likewise, low public support for increasing the price of alcohol may indicate poor public awareness of the effectiveness of price controls in reducing alcohol-related harm.

Five studies found very varied levels of support for increasing the rate of excise duty on "alcopops". These ready-to-drink spirits were singled out by the Federal Government for taxation reform in 2008 due to their perceived popularity with underage drinkers and because they had previously escaped taxation at equivalent rates to other spirits [66]. Two polls of the Australian public conducted by public health organisations, found high levels of support for increasing the tax on alcopops if the revenue was dedicated towards prevention and treatment of alcohol-related harms (73-84% supported) [52,65]. However, a conflicting result was found in three polls commissioned by a peak alcohol industry organisation, sampling the same target population at a similar point in time. Rather than directly measuring support, these polls assessed public preference between two policy responses, and found consistent high levels of opposition to the alcopops tax in favour of a "more comprehensive strategy" to tackle binge drinking (77-81% opposed) [38-40] (see Additional file 5). Hence, the polarised responses for this particular policy are likely explained by the variation in the wording, style and intent of the survey questions.

## Discussion

This review of public opinion towards alcohol control policy has found that majority support exists for most of the regulatory controls proposed by the National Preventative Health Taskforce in their Alcohol Strategy for Australia [13], but revealed wide variation in levels of support between the different types of controls.

Australians are most supportive of stricter monitoring and enforcement of licensees' compliance with liquor control legislation, the inclusion of health warnings on alcohol labelling, restrictions on alcohol advertising, and targeted controls on high risk venues and populations. Controls which restrict availability of alcohol using price, tax, reduced trading hours or reduced outlet density receive less support. This distinction in support is similar to previously documented observations of public opinion towards alcohol controls in other Western countries, which found that support is lower for policies which are designed to reduce availability and higher for policies concerning information, education or treatment for targeted individuals [19-22].

Further comparison of these controls by differing level of support reveals another point of distinction: the Australian public are most supportive of controls that are distal to individuals (such as regulation of licensees or alcohol promotions), and least supportive of proximal controls (such as regulation of price and availability) which can be more readily perceived to impact directly on individual drinking behaviour. This division in levels of support between controls on 'others' versus controls on 'self' suggests that the Australian public do recognise the problems with alcohol and accept that some intervention is required, but do *not *recognise the problem to be shared by the majority of the 'moderate' drinking population.

How narrowly the public recognise the problem with alcohol will in turn impact on who they believe should be responsible for providing solutions and to who these solutions should be targeted. A public that believes that the problem with alcohol is with only a small minority and whose core beliefs centre on individual self-reliance, will resist government intervention and be more supportive of targeted interventions than universal controls. On the other hand, a public that recognises that the problems with alcohol are beyond the scope of the individual and more a product of the social and economic environment in which we live, will expect the government to intercede and lend higher support to a combination of both universal and targeted controls.

At the outset of this review, greater awareness of alcohol-related harm and increased regulatory controls of alcohol in the present day, invited comparison with attitudes and the regulatory framework for tobacco in the past. It was hypothesised that opinions towards alcohol controls could be following trends similar to those observed towards tobacco controls during the past three decades.

### Is public support for alcohol controls increasing over time?

Among the various sources of data included in this review, only data obtained from the NDSHS was suitable for comparing levels of support over time. It was difficult to make direct links and identify prevalence trends between the remaining data because the data were sourced from a wide variety of sample populations and study designs, using different question wording.

Analysis by Wilkinson *et al *of public opinion towards alcohol control policy measured in the NDSHS between 1993 and 2004 found that public support was gradually decreasing throughout this period in all but two policy areas from a total of sixteen [18]. Since the Wilkinson review, results from the NDSHS conducted in 2007 have been released. Between 2004 and 2007, public support increased for eleven of the sixteen alcohol control policies examined (the increase was statistically significant for eight policy items) [8]. Wherever a decrease in support was observed, this decrease was not found to be statistically significant [8]. It is too early to tell whether this changing direction of support is part of new trend or just a temporary levelling of the existing trend.

Comparable to the experience in Ontario [25], the period of decreasing public support in Australia coincided with an era of increasing liberalisation of alcohol controls. Likewise, the gains in public support for alcohol controls observed between 2004 and 2007 have occurred at a time when governments are beginning to tighten previously loosened reins on alcohol. The critical issue derived from this is whether public opinion towards alcohol policy has followed the lead of regulators, or has alcohol control policy responded to public attitudes?

### Does public support for alcohol controls increase post-implementation?

Two studies measured community support for additional liquor licensing restrictions in Indigenous communities before and after implementation [53,54]. Both studies found that support for the new controls increased uniformly after a period of implementation, suggesting that similar to the experience with tobacco [31], public acceptability of alcohol controls increases once their impact has been personally experienced and found to be less disruptive (or more beneficial) than initially anticipated.

Random breath testing (RBT) of motorists is an earlier example of an alcohol control measure for which public support increased following implementation. Public support for RBT in NSW was 64% prior to its introduction in 1982 and increased to 85% twelve months after its implementation [67]. Nation-wide, RBT programs became more visible, intensive and rigorously enforced during the early 1990s [68]; during this period Australian's support for RBT increased from 88% in 1986 to 97% in 1992 and has been maintained at these high levels ever since [69-71].

This knowledge should encourage governments to implement new alcohol policy without fear of a backlash even when public support is initially low, because support will likely increase following implementation. In response to the rhetorical question posed above, this finding adds more strength to the position that public opinion is influenced by policy and the contextual environment to which policy gives shape.

### Is public support higher for alcohol controls which seek to protect children and innocent third parties from harm?

From tobacco control, we have learnt that the public are more likely to support government intervention in controversial areas if they act in the interests of children [27,33,34]. This review provides some evidence of differential support for regulation of alcohol promotions between controls to protect children in comparison to universal controls. Public support for banning alcohol advertising visible within 1 km of schools is greater than double the level of support given for banning advertising of alcohol entirely [46,47]. Support for restricting advertising of alcohol to times and places that minimise exposure to children is higher than support for widespread advertising restrictions [6,41,46,48,61].

Higher public support in tobacco control also extended to regulations protecting innocent third parties from environmental tobacco smoke [31]. This review found that the public are more supportive of controls targeting late night venues than controls which apply to all licensed premises. Given the well documented association between late night venues and alcohol-related violence [72], one possible explanation for this higher support may be that the public are more accepting of intervention because they recognise that these controls are designed to increase safety.

For policy makers, these findings imply that controls directed towards protecting children from exposure to alcohol promotions and innocent bystanders from the effects of passive drinking may be a popular starting point from which to begin alcohol policy reform.

### Sources of public opinion data

The public opinion data included in this review were sourced from diverse jurisdictions and populations at one point in time, using varied survey designs. The implication of this diversity was that only broad comparisons, rather than specific evaluations or meta-analysis were possible during analysis.

The precision and validity of public opinion data is called to question at times over the influence that survey design features may exert on the responses achieved; question wording, ordering of questions and alternative responses, and the inclusion of value-laden words or phrases all may exert an influence on the respondent [59]. Consider the question wording used to elicit the polarised levels of support for the alcopops tax between five studies in this review. Highlighting the potential use of the additional tax for the prevention of emotive poor health outcomes such as cancer and heart disease may have had bearing on the high levels of support in favour of the alcopops tax [52]. Whereas pitching the alcopops tax against an alternative but unknown "wider and more comprehensive" policy to address binge-drinking, using language which implies that the alternative policy is superior to the tax, and highlighting that others oppose the tax, resulted in equally high levels of opposition to the tax [38-40].

Many studies included in this review neglected to publish the question wording used to measure the presented public opinion, inhibiting a critical analysis of the published result. Similarly, critical analysis of some included results was further constrained because research methodology details sought during data extraction were incompletely reported.

Future public opinion researchers should strive to improve their reporting to increase the legitimacy of public opinion data and enhance the potential for drawing comparisons between public opinion data within and across jurisdictions and over time. In particular, consideration should be given to replicating previous study designs and using consistent question phrasing. The STROBE Statement is a useful checklist for researchers aiming to increase the transparency of their data reporting [73].

## Conclusions

Public opinion towards alcohol controls in Australia is varied. Governments are well supported to tighten alcohol controls targeted towards alcohol promotions, licensees or marginalised "problem" drinkers. However, controls which impact on the availability of alcohol across the population are unpopular. Given the inextricable link between availability and alcohol-related harm, unpopularity is not enough to warrant inaction in this regulatory area. Evidence suggests that those who are most likely to oppose alcohol controls are the proportion of the population who are most likely to experience alcohol-related harm due to high-risk drinking behaviours [18].

This review has identified some potential inroads to introducing universal alcohol policy reform which will be accepted by the Australian public. Alcohol controls which are framed as protecting children from exposure to alcohol promotions or innocent third parties from harm associated with "passive drinking" are unlikely to be opposed and so may be a good starting point for policy reform. A factor which may induce higher support is when the intent of new policies is well understood and the rationale for change is explicit to the public. Policy advocates and decision makers should utilise public opinion data as a tool to reveal gaps in public understanding of new policies and respond to identified knowledge deficits by matter of routine during the policy development process. In instances when the effectiveness of an alcohol control is well established in the research literature, but public support is low, public health advocates should explore strategies for translating research into formats and forums which are meaningful and accessible to the public. It is equally important that public opinion research findings and policy implications are accurately and succinctly communicated to policy makers. Finally, an increasing regulatory environment is likely to influence public attitudes to alcohol controls. As further policy is implemented and experienced by the public, there is some evidence to suggest that support will increase and new controls will be accepted.

## List of abbreviations

CATI: Computer Assisted Telephone Interviewing; D&C: Drop and Collect; NDSHS: National Drug Strategy Household Survey; NSW: New South Wales; NT: Northern Territory; RBT: Random Breath Testing; WA: Western Australia;

## Competing interests

The authors declare that they are not connected, through funding or other mutual interests, with the tobacco, alcohol, pharmaceutical or gaming industries.

This work was completed while RM held the position of Chair, Preventive Health Taskforce, and CT was conducting research to support the development of the National Preventative Health Strategy.

## Authors' contributions

CT performed the review and analysis and drafted the manuscript. RM conceived the research question, supervised the research and reviewed the manuscript. CL contributed to the analysis and reviewed the manuscript. All authors read and approved the final manuscript.

## Pre-publication history

The pre-publication history for this paper can be accessed here:

http://www.biomedcentral.com/1471-2458/11/58/prepub

## Supplementary Material

Additional file 1 **Search strategy example**. Click here for file

Additional file 2 **Public support for liquor control regulations**. Click here for file

Additional file 3 **Public support for regulation of alcohol promotions**. Click here for file

Additional file 4 **Public support for reforming alcohol taxation and pricing**. Click here for file

Additional file 5 **DSICA Galaxy Poll 2009 – ‘Alcopops Tax’ Survey**. Click here for file
