# Supplementary material for: A review of public opinion towards alcohol controls in Australia
Source: BMC Public Health. 2011 Jan 27;11:58. doi: 10.1186/1471-2458-11-58 (PMC3048532; doi:10.1186/1471-2458-11-58)
Supplement: Additional file 1 — Search strategy example. [file 1471-2458-11-58-S1.DOC]

**Additional File 1: Search strategy using** MEDLINE (ISI)

| # 6 | [117](http://apps.isiknowledge.com.ezproxy.lib.monash.edu.au/summary.do?product=MEDLINE&doc=1&qid=14&SID=Y1piD@cf3eoIMMM8eD7&search_mode=CombineSearches) | #5 AND #4  Databases=In-Process, MEDLINE Timespan=1998-2009 |
| --- | --- | --- |
| # 5 | [>100,000](http://apps.isiknowledge.com.ezproxy.lib.monash.edu.au/summary.do?product=MEDLINE&doc=1&qid=13&SID=Y1piD@cf3eoIMMM8eD7&search_mode=GeneralSearch) | MeSH Heading=(alcohol) OR(((Topic=(alcohol) OR Topic=(drinking)) OR Topic=( binge-drinking )) OR Topic=( binge drinking ))  Databases=In-Process, MEDLINE Timespan=1998-2009 |
| # 4 | [2,214](http://apps.isiknowledge.com.ezproxy.lib.monash.edu.au/summary.do?product=MEDLINE&doc=1&qid=4&SID=Y1piD@cf3eoIMMM8eD7&search_mode=CombineSearches) | #3 AND #2 AND #1  Databases=In-Process, MEDLINE Timespan=1998-2009 |
| # 3 | [>100,000](http://apps.isiknowledge.com.ezproxy.lib.monash.edu.au/summary.do?product=MEDLINE&doc=1&qid=3&SID=Y1piD@cf3eoIMMM8eD7&search_mode=GeneralSearch) | (((((((Topic=(regulat*) OR Topic=(policy)) OR Topic=(legislat*)) OR Topic=(tax*)) OR Topic=(pric*)) OR Topic=(ban)) OR Topic=(restrict*)) OR Topic=(control)) OR Topic=(interven*)  Databases=In-Process, MEDLINE Timespan=1998-2009 |
| # 2 | [>100,000](http://apps.isiknowledge.com.ezproxy.lib.monash.edu.au/summary.do?product=MEDLINE&doc=1&qid=2&SID=Y1piD@cf3eoIMMM8eD7&search_mode=GeneralSearch) | ((((((((((((Topic=( public opinon ) OR Topic=( public opinions )) OR Topic=( public attitude )) OR Topic=( public attitudes )) OR Topic=( public perception )) OR Topic=( public perceptions )) OR Topic=( community opinion )) OR Topic=( community opinions )) OR Topic=( community attitude )) OR Topic=( community attitiudes )) OR Topic=( community perception )) OR Topic=( community perceptions )) OR MeSH Heading=(attitude*)) OR((Topic=(opinion*) OR Topic=(attitude*)) OR Topic=(perception*))  Databases=In-Process, MEDLINE Timespan=1998-2009 |
| # 1 | [47,888](http://apps.isiknowledge.com.ezproxy.lib.monash.edu.au/summary.do?product=MEDLINE&doc=1&qid=1&SID=Y1piD@cf3eoIMMM8eD7&search_mode=GeneralSearch) | Topic=(Australia*)  Databases=In-Process, MEDLINE Timespan=1998-2009 |
