# Supplementary material for: A review of public opinion towards alcohol controls in Australia
Source: BMC Public Health. 2011 Jan 27;11:58. doi: 10.1186/1471-2458-11-58 (PMC3048532; doi:10.1186/1471-2458-11-58)
Supplement: Additional file 2 — Public support for liquor control regulations. [file 1471-2458-11-58-S2.DOC]

| **Control** | **Level of support** | **Survey question** | **Jurisdiction (year) data collected**  **Population sampled**  **Sample size (response)**  **Method of data collection** | **Source** |
| --- | --- | --- | --- | --- |
| **STRONGER ENFORCEMENT / PENALTIES FOR LICENSEES** | | | | |
| Stricter laws against serving drunk customers | **83.3% in 2007**  **83.8% in 2004**  **85% in 2001** | *To reduce the problems associated with excessive alcohol use, to what extent would you support or oppose stricter enforcement of the law against serving customers who are drunk?* | Australia (2001-2007)  Aged 14yrs +  2007 n=23,455 (49.3%)  2004 n=29,455 (45.6%)  2001 n=26,744 (50%)  CATI / D&C | AIHW 2008 [41]  AIHW 2005 [42]  AIHW 2002 [43] |
|  | 80.1% in NT – 84.2% in NSW | *as above* | States & territories (2007)  Aged 14 yrs +  n=23,455 (49.3%)  CATI / D&C | AIHW 2008 [44] |
| Increasing RSA checks by licensing authorities | 79.7% | *n/a* | Rural NSW (2007)  Adults aged 18 yrs +  n=200 (34.9%)  Self-completed postal | Hawkins et al 2009 [36] |
| Increasing RSA checks by police | 82.3% | *n/a* | Rural NSW (2007)  Adults aged 18 yrs +  n=200 (34.9%)  Self-completed postal | Hawkins et al 2009 [36] |
| Increasing RSA fines and penalties | 75.6% | *n/a* | Rural NSW (2007)  Adults aged 18 yrs +  n=200 (34.9%)  Self-completed postal | Hawkins et al 2009 [36] |
| **INCREASE LATE NIGHT SAFETY** | | | | |
| Strict monitoring late night licensed premises | **75.2% in 2007**  **72.1% in 2004**  **60.0% in 2001** | *To reduce the problems associated with excessive alcohol use, to what extent would you support or oppose strict monitoring of late night licensed premises?* | Australia (2001-2007)  Aged 14yrs +  2007 n=23,455 (49.3%)  2004 n=29,455 (45.6%)  2001 n=26,744 (50%)  CATI / D&C | AIHW 2008 [41]  AIHW 2005 [42]  AIHW 2002 [43] |
|  | 69.8% in NT – 77% in Vic | *as above* | States & territories (2007)  Aged 14 yrs +  n=23,455 (49.3%)  CATI / D&C | AIHW 2008 [44] |
| Restricting late night alcohol trading | **58.0% in 2007**  **51.9% in 2004**  **50.9% in 2001** | *To reduce the problems associated with excessive alcohol use, to what extent would you support or oppose restricting late night trading of alcohol?* | Australia (2001-2007)  Aged 14yrs +  2007 n=23,455 (49.3%)  2004 n=29,455 (45.6%)  2001 n=26,744 (50%)  CATI / D&C | AIHW 2008 [41]  AIHW 2005 [42]  AIHW 2002 [43] |
|  | 50.6% in WA – 61.9% in NSW | *as above* | States & territories (2007)  Aged 14 yrs +  n=23,455 (49.3%)  CATI / D&C | AIHW 2008 [44] |
| “Lockout” times for licensed premises | 71.4% | *n/a* | Rural NSW (2007)  Adults aged 18 yrs +  n=200 (34.9%)  Self-completed postal | Hawkins et al 2009 [36] |
| Staggered closing times of licensed premises | 55.8% | *n/a* | Rural NSW (2007)  Adults aged 18 yrs +  n=200 (34.9%)  Self-completed postal | Hawkins et al 2009 [36] |
| **REDUCE AVAILABILITY** | | | | |
| Reducing alcohol trading hours for pubs and clubs | **38.9% in 2007**  **32.0% in 2004**  **32.4% in 2001**  **35.0% in 1998** | *To reduce the problems associated with excessive alcohol use, to what extent would you support or oppose reducing trading hours for all pubs and clubs?* | Australia (1998-2007)  Aged 14yrs +  2007 n=23,455 (49.3%)  2004 n=29,455 (45.6%)  2001 n=26,744 (50%)  1998 n=10,030 (56%)  CATI / D&C | AIHW 2008 [41]  AIHW 2005 [42]  AIHW 2002 [43]  Adhikari & Summerill 1998 [45] |
|  | 28.1% in WA – 44% in NSW in 2007 | *as above* | States & territories (2007)  Aged 14 yrs +  n=23,455 (49.3%)  CATI / D&C | AIHW 2008 [44] |
|  | 48.5% | *n/a* | Rural NSW (2007)  Adults aged 18 yrs +  n=200 (34.9%)  Self-completed postal | Hawkins et al 2009 [36] |
| Reducing alcohol outlet density | **32.2% in 2007**  **28.5% in 2004**  **28.7% in 2001**  **34.3% in 1998** | *To reduce the problems associated with excessive alcohol use, to what extent would you support or oppose reducing the number of outlets that serve alcohol?* | Australia (1998-2007)  Aged 14yrs +  2007 n=23,455 (49.3%)  2004 n=29,455 (45.6%)  2001 n=26,744 (50%)  1998 n=10,030 (56%)  CATI / D&C | AIHW 2008 [41]  AIHW 2005 [42]  AIHW 2002 [43]  Adhikari & Summerill 1998 [45] |
|  | 26.9% in WA – 43.2% in NT | *as above* | States & territories (2007)  Aged 14 yrs +  n=23,455 (49.3%)  CATI / D&C | AIHW 2008 [44] |
|  | 39.7% | *n/a* | Rural NSW (2007)  Adults aged 18 yrs +  n=200 (34.9%)  Self-completed postal | Hawkins et al 2009 [36] |
| Increasing alcohol-free dry zones | **62.5% in 2007**  **63.3% in 2004**  **65.7% in 2001**  **68.6% in 1998** | *To reduce the problems associated with excessive alcohol use, to what extent would you support or oppose increasing the number of alcohol-free zones or dry areas?* | Australia (1998-2007)  Aged 14yrs +  2007 n=23,455 (49.3%)  2004 n=29,455 (45.6%)  2001 n=26,744 (50%)  1998 n=10,030 (56%)  CATI / D&C | AIHW 2008 [41]  AIHW 2005 [42]  AIHW 2002 [43]  Adhikari & Summerill 1998 [45] |
|  | 56.4% in NT – 66.0% in NSW | *as above* | States & territories (2007)  Aged 14 yrs +  n=23,455 (49.3%)  CATI / D&C | AIHW 2008 [44] |
|  | 76.8% | *n/a* | Rural NSW (2007)  Adults aged 18 yrs +  n=200 (34.9%)  Self-completed postal | Hawkins et al 2009 [36] |
| **LOCAL INITIATIVES IN INDIGENOUS COMMUNITIES** | | | | |
| Restricting bar trading hours on weekdays | **44.4% in 2003**  **37.9% in 2002** | *Do you know that pubs aren’t allowed to sell anything but light beer before half past eleven in the morning on weekdays? Did you like the idea when it first came in/ do you like it now?* | Alice Springs (2002-03)  Residents/visitors 19 Aboriginal town camps, aged 18yrs +  n=277  Interviews | Tangentyere Council 2003 [54] |
| Reducing bar trading hours | 55-70% | *n/a* | Tennant Creek (1998)  Aged 18yrs +  n=271  Interviews | Gray et al 2000 [53] |
| Reducing “takeaway” trading hours on weekdays | **59.6% in 2003**  **50.9% in 2002** | *Did you know that, before you could buy take-away grog at 12 o’clock but that they changed it to 2 o’clock? Did you like the idea when it first came in/ do you like it now?* | Alice Springs (2002-03)  Residents/visitors 19 Aboriginal town camps, aged 18yrs +  n=277  Interviews | Tangentyere Council 2003 [54] |
| Reducing takeaway trading hours | 59-71% | *n/a* | Tennant Creek (1998)  Aged 18yrs +  n=271  Interviews | Gray et al 2000 [53] |
| Ban takeaway sale of alcohol in containers >2L | **51.3% in 2003**  **48.0% in 2002** | *Do you know that you can’t buy five litres of grog any more, only two litres? Did you like the idea when it first came in/ do you like it now?* | Alice Springs (2002-03)  Residents/visitors 19 Aboriginal town camps, aged 18yrs +  n=277  Interviews | Tangentyere Council 2003 [54] |
| Restricting alcohol container size | 55-76% | *n/a* | Tennant Creek (1998)  Aged 18yrs +  n=271  Interviews | Gray et al 2000 [53] |
| Require food with alcohol sales | 66-86% | *n/a* | Tennant Creek (1998)  Aged 18yrs +  n=271  Interviews | Gray et al 2000 [53] |
