# Supplementary material for: A review of public opinion towards alcohol controls in Australia
Source: BMC Public Health. 2011 Jan 27;11:58. doi: 10.1186/1471-2458-11-58 (PMC3048532; doi:10.1186/1471-2458-11-58)
Supplement: Additional file 3 — Public support for regulation of alcohol promotions. [file 1471-2458-11-58-S3.DOC]

| **Control** | **Level of support** | **Survey question** | **Jurisdiction (year) data collected**  **Population sampled**  **Sample size (response)**  **Method of data collection** | **Source** |
| --- | --- | --- | --- | --- |
| **GOVERNMENT REGULATION OF ALCOHOL ADVERTISING** | | | | |
| Government regulation of marketing and advertising of alcohol products | 67% | *n/a* | Australia (2009)  Aged 18yrs +  n=1,030  CATI | AERF 2009 [15] |
| Alcohol advertising reviewed by independent authority | 77% | *How strongly do you agree/disagree that “alcohol advertising should be reviewed by an independent authority before appearing in the media like TV and press?”* | Australia (2007)  Aged 18yrs +  n=1,054  Online survey | Tinworth 2008 [6] |
|  | 80% | *“The regulation of alcohol advertising and marketing is largely self regulated by the alcohol industry. Would you support or oppose the introduction of alcohol advertising being reviewed by an independent authority before appearing in media like TV, magazines and newspapers?”* | Victoria (2009)  Aged 16yrs +  n=1523  CATI | APC 2009 [46] |
| **STRONGER RESTRICTIONS ON ALCOHOL ADVERTISING** | | | | |
| Alcohol advertising reduced OR banned | 70% | *n/a* | Australia (2008)  Aged 14yrs+  n=653  CATI | Salvation Army 2008 [61] |
| Government ban on ALL alcohol advertising and sponsorship | 38% | *To what extent do you agree…”the government should ban all alcohol advertising and sponsorship”* | Western Australia (2009)  Aged 18yrs+  n=800  CATI | Scerri et al 2009 [47] |
| Stronger restrictions on alcohol advertising | 60% | *Nominate which best describes your attitude: “the advertising of drinks should be banned entirely; should be restricted more than it is but not entirely banned; is quite acceptable as it is; should be less restricted than it seems to be at present; other.”* | Australia (2005)  Aged 18yrs+  n=1000  CATI | King et al 2005 [48] |
| **REDUCE YOUNG PEOPLE’S EXPOSURE TO ALCOHOL ADVERTISING** | | | | |
| Limit TV advertising of alcohol until after 9.30pm | **72.2% in 2007**  **71.4% in 2004**  **69.5% in 2001**  **72.7% in 1998** | *To reduce the problems associated with excessive alcohol use, to what extent would you support or oppose limiting the advertising for alcohol on TV after 9.30pm?* | Australia (1998-2007)  Aged 14yrs +  2007 n=23,455 (49.3%)  2004 n=29,455 (45.6%)  2001 n=26,744 (50%)  1998 n=10,030 (56%)  CATI / D&C | AIHW 2008 [41]  AIHW 2005 [42]  AIHW 2002 [43]  Adhikari & Summerill 1998 [45] |
|  | 64.1% in NT – 74.2% in Tas | *as above* | States & territories (2007)  Aged 14 yrs +  n=23,455 (49.3%)  CATI / D&C | AIHW 2008 [44] |
| Restrict alcohol advertising to minimise exposure to people under 18yrs of age | 82% | *Do you agree or disagree… “advertisements for alcoholic products should be restricted so that they are less likely to be seen by people under 18 years of age”?* | Victoria (2009)  Aged 16yrs +  n=1523  CATI | APC 2009 [46] |
|  | 77% | *How strongly do you agree/disagree that “alcohol advertising should be restricted to locations, publications, and times that are less likely to be seen by people under 18 years of age?”* | Australia (2007)  Aged 18yrs +  n=1,054  Online survey | Tinworth 2008 [6] |
| Ban alcohol advertising within 1km of schools | 78% | *Do you agree or disagree that “advertising alcohol on billboards should be banned within one kilometre of schools”?* | Victoria (2009)  Aged 16yrs +  n=1523  CATI | APC 2009 [46] |
| **BANNING ALCOHOL SPONSORSHIP OF SPORTING EVENTS** | | | | |
| Banning alcohol sponsorship of sporting events | **48.5% in 2007**  **46.1% in 2004**  **43.9% in 2001**  **44.8% in 1998** | *To reduce the problems associated with excessive alcohol use, to what extent would you support or oppose banning alcohol sponsorship of sporting events?* | Australia (1998-2007)  Aged 14yrs +  2007 n=23,455 (49.3%)  2004 n=29,455 (45.6%)  2001 n=26,744 (50%)  1998 n=10,030 (56%)  CATI / D&C | AIHW 2008 [41]  AIHW 2005 [42]  AIHW 2002 [43]  Adhikari & Summerill 1998 [45] |
|  | 36.3% in NT – 53.6% in WA | *as above* | States & territories (2007)  Aged 14 yrs +  n=23,455 (49.3%)  CATI / D&C | AIHW 2008 [44] |
|  | 45% | *To what extent do you agree…”the government should ban all alcohol advertising and sponsorship during sports games (on TV/sports grounds)”* | Western Australia (2009)  Aged 18yrs+  n=800  CATI | Scerri et al 2009 [47] |
| Reduce exposure to advertising linking alcohol and sport on television during family viewing time (before 9pm) | 73% | *n/a* | Australia (2009)  Aged 18yrs +  n=1,030  CATI | AERF 2009 [62] |
| Ban alcohol advertising on sporting arenas which is visible on television | 59% | *n/a* | Australia (2009)  Aged 18yrs +  n=1,030  CATI | AERF 2009 [62] |
| **REQUIRE HEALTH ADVISORY & OTHER LABELLING ON ALCOHOL PACKAGING** | | | | |
| Health warnings on alcohol containers | **69% in 2006**  **69% in 2001** | *In your opinion should all alcohol products carry a health warning on the pack or bottle just like cigarettes?* | Australia (2001, 2006)  Aged 14yrs+  2001 n=475  CATI | Roy Morgan Research 2001 [63]  Salvation Army 2006 [64] |
|  | 89% | *Do you support or oppose the introduction of labels on alcoholic beverage containers which detail… a warning message advising that exceeding the recommended guidelines may be harmful?* | Victoria (2009)  Aged 16yrs +  n=1523  CATI | VicHealth 2009 [49] |
| Targeted advice for specific groups on alcohol containers | 91% | *Do you support or oppose the introduction of labels on alcoholic beverage containers which detail… health warnings for specific groups eg pregnant women, young people?* | Victoria (2009)  Aged 16yrs +  n=1523  CATI | VicHealth 2009 [49] |
| Increase the size of standard drink labels on alcohol containers | **65.8% in 2007**  **66.4% in 2004**  **67.9% in 2001** | *To reduce the problems associated with excessive alcohol use, to what extent would you support or oppose increasing the size of standard drink labels on alcohol containers?* | Australia (2001-2007)  Aged 14yrs +  2007 n=23,455 (49.3%)  2004 n=29,455 (45.6%)  2001 n=26,744 (50%)  CATI / D&C | AIHW 2008 [41]  AIHW 2005 [42]  AIHW 2002 [43] |
|  | 58.5% in NT – 67% in Vic | *as above* | States & territories (2007)  Aged 14 yrs +  n=23,455 (49.3%)  CATI / D&C | AIHW 2008 [44] |
| Require national drinking guidelines on alcohol containers | **70.9% in 2007**  **69.9% in 2004**  **71.0% in 2001** | *To reduce the problems associated with excessive alcohol use, to what extent would you support or oppose requiring information on national drinking guidelines on all alcohol containers?* | Australia (2001-2007)  Aged 14yrs +  2007 n=23,455 (49.3%)  2004 n=29,455 (45.6%)  2001 n=26,744 (50%)  CATI / D&C | AIHW 2008 [41]  AIHW 2005 [42]  AIHW 2002 [43] |
|  | 59.9% in NT – 71.6% in NSW | *as above* | States & territories (2007)  Aged 14 yrs +  n=23,455 (49.3%)  CATI / D&C | AIHW 2008 [44] |
|  | 85% | *Do you support or oppose the introduction of labels on alcoholic beverage containers which detail… recommended daily guidelines for low risk alcohol consumption?* | Victoria (2009)  Aged 16yrs +  n=1523  CATI | VicHealth 2009 [49] |
| Include ingredient and nutritional labelling on alcohol containers | 86% (ingredient)  76% (nutritional) | *Do you support or oppose the introduction of labels on alcoholic beverage containers which detail… ingredients/ nutritional information (energy, protein, fat, carbs, sugars) displayed on labels?* | Victoria (2009)  Aged 16yrs +  n=1523  CATI | VicHealth 2009 [49] |
|  | 81% (ingredient)  75% (nutritional) | *How strongly do you agree or disagree that “it should be a requirement that the ingredients in alcoholic beverages/ nutritional information (e.g. the amount of sugar and kilojoules are displayed on the bottle/can/cask?* | Australia (2007)  Uni students aged 17yrs+  n=7237 (56%)  Web-based survey | Kypri et al 2007 [37] |
| **COUNTER ADVERTISING** | | | | |
| Health warnings included in print media alcohol advertisements | 70% | *n/a* | Australia (2006)  Aged 14yrs+  CATI | Salvation Army 2006 [64] |
| Health warnings included in TV alcohol advertisements | 74% | *n/a* | Australia (2006)  Aged 14yrs+  CATI | Salvation Army 2006 [64] |
