# Supplementary material for: A review of public opinion towards alcohol controls in Australia
Source: BMC Public Health. 2011 Jan 27;11:58. doi: 10.1186/1471-2458-11-58 (PMC3048532; doi:10.1186/1471-2458-11-58)
Supplement: Additional file 4 — Public support for reforming alcohol taxation and pricing. [file 1471-2458-11-58-S4.DOC]

| **Control** | **Level of support** | **Survey question** | **Jurisdiction (year) data collected**  **Population sampled**  **Sample size (response)**  **Method of data collection** | **Source** |
| --- | --- | --- | --- | --- |
| **INCREASE THE PRICE OF ALCOHOL** | | | | |
| Increase the price of alcohol | **24.1% in 2007**  **20.9% in 2004**  **20.5% in 2001**  **26.6% in 1998** | *To reduce the problems associated with excessive alcohol use, to what extent would you support or oppose increasing the price of alcohol?* | Australia (1998-2007)  Aged 14yrs +  2007 n=23,455 (49.3%)  2004 n=29,455 (45.6%)  2001 n=26,744 (50%)  1998 n=10,030 (56%)  CATI / D&C | AIHW 2008 [41]  AIHW 2005 [42]  AIHW 2002 [43]  Adhikari & Summerill 1998 [45] |
|  | 21.7% in NT – 26.3% in NSW | *as above* | States & territories (2007)  Aged 14 yrs +  n=23,455 (49.3%)  CATI / D&C | AIHW 2008 [44] |
| Set a minimum sale price for alcoholic beverages sold in bottles or containers | 79% | *Do you agree or disagree? The government should set a minimum selling price for alcoholic beverages sold in bottles or other containers to ensure that alcohol is not cheaper than bottled water or soft drinks.* | Victoria (2009)  Aged 16yrs +  n=1523  CATI | APC 2009 [50] |
| **INCREASE THE TAX ON ALCOHOL** | | | | |
| Increase the tax on alcohol and dedicate revenue for prevention and treatment of alcohol-related harms | **41.3% in 2007**  **38.6% in 2004** | *To reduce the problems associated with excessive alcohol use, to what extent would you support or oppose increasing the tax on alcohol products to pay for health, education, and the cost of treating alcohol related problems?* | Australia (2004, 2007)  Aged 14yrs +  2007 n=23,455 (49.3%)  2004 n=29,455 (45.6%)  CATI / D&C | AIHW 2008 [41]  AIHW 2005 [42] |
|  | 35.2% in NT – 43.5% in NSW | *as above* | States & territories (2007)  Aged 14 yrs +  n=23,455 (49.3%)  CATI / D&C | AIHW 2008 [44] |
|  | 51% | *Would you support an additional charge (say around 2% or 10 cents) being levied on alcohol products to help fund alcohol treatment and prevention services?* | Australia (2007)  Aged 18yrs +  n=1,054  Online survey | Tinworth 2006 [6] |
|  | 67% | *Do you agree or disagree? An additional tax should be levied on alcohol products to fund more alcohol treatment and education programs.* | Victoria (2009)  Aged 16yrs +  n=1523  CATI | APC 2009 [50] |
| Increase tax on alcohol to reduce consumption | 41% | *Would you support or oppose “placing additional taxes on alcoholic drinks to reduce their consumption?”* | Victoria (2006)  Aged 18yrs +  n=1000  CATI | Pennay & Bateman 2007 [51] |
| Set tax rate on alcohol according to alcohol content per volume | 67% | *Do you agree or disagree? The tax rate on alcohol should be set according to how much alcohol the product contains.* | Victoria (2009)  Aged 16yrs +  n=1523  CATI | APC 2009 [50] |
| Set the highest tax rate on alcohol for products that cause the most harm | 61% | *Do you agree or disagree? The tax rate should be highest for alcoholic products (such as bottled spirits and cask wine) that cause the most problems (eg. violence).* | Victoria (2009)  Aged 16yrs +  n=1523  CATI | APC 2009 [50] |
| **INCREASE THE TAX ON ALCOPOPS** | | | | |
| Vote down alcopops tax in favour of a wider and more comprehensive strategy to tackle binge drinking | **78% in 05/2009**  **77% in 01/2009**  **81% in 07/2008** | *Even though the Government is to collect over $1.6billion with this so-called ‘Alcopops Tax’, some people have suggested that it should be voted down again by the Senate in favour of a wider and more comprehensive strategy to tackle binge-drinking among young people – do you agree or disagree with this suggestion?* | Australia (2008-2009)  Aged 18yrs +  05/2009 n=1046  01/2009 n=1058  07/2008 n=1042  CATI | DSICA 2008 [38]  DSICA 2009 [39]  DSICA 2009 [40] |
| Increase tax on alcopops to equate with tax on normal spirits | 57% | *Would you personally be in favour or against an increase in tax on alcopops to bring it in line with the tax on normal spirits?* | Australia (2008)  Aged 18yrs +  n=1202  CATI | ASH et al 2008 [52] |
| Increase tax on alcopops and dedicate revenue for prevention and treatment of alcohol-related harm | 84% | *Would you personally be in favour or against an increase in the tax on alcopops if most of the extra tax is used for health programs to prevent diseases such as heart disease and cancer?* | Australia (2008)  Aged 18yrs +  n=1202  CATI | ASH et al 2008 [52] |
| Dedicate revenue collected from alcopops tax for prevention and treatment of alcohol-related harms | 84% (prevention)  73% (treatment) |  | Australia (2008)  Aged 18yrs +  n=1046  CATI | AERF 2008 [65] |
