# Supplementary material for: A review of public opinion towards alcohol controls in Australia
Source: BMC Public Health. 2011 Jan 27;11:58. doi: 10.1186/1471-2458-11-58 (PMC3048532; doi:10.1186/1471-2458-11-58)
Supplement: Additional file 5 — DSICA Galaxy Poll 2009 – ‘Alcopops Tax’ Survey. [file 1471-2458-11-58-S5.PDF]

TOPLINE RESULTS	
Subject:	**DSICA Galaxy Poll 2009 – 'Alcopops Tax' Survey**
Date:	**Monday 4th May 2009**

Summary Methodology

This research project was conducted by Galaxy Research on behalf of the Distilled Spirits Industry Council of Australia (DSICA).

The study involved a quantitative survey of a sample of Australian adults aged 18 years and over, and was conducted using the Galaxy Omnibus on the weekend of 1st – 3rd May 2009.

Interviews were conducted using CATI (Computer Aided Telephone Interviewing), with telephone numbers randomly selected from the electronic White Pages, i.e. a probability sample allowing statistical tests of significance.

Minimum age, gender and region quotas were set, and following completion of interviewing the data was weighted by age, gender and region to reflect the latest ABS population estimates – these measures ensuring accurate representation.

The total sample comprised n=1,046 respondents, with geographical representation as follows:

- New South Wales/Australian Capital Territory: n=310.
- Victoria/Tasmania: n=289.
- Queensland: n=195.
- South Australia: n=125.
- Western Australia: n=127.

The total sample size yields results accurate to within a maximum margin of error of +/-3.0% at the 95% confidence interval. In simple terms this means that we can be 95% sure that individual survey findings are accurate to within this maximum margin or error when falling around the 50% mark, with the error reducing significantly as individual results move away from the 50% mark. However, results for sub-samples, such as geo-demographic groups are likely to have larger margins of error associated with them.

Wherever appropriate the results of this study have been compared against two similar surveys conducted in late July 2008 and mid January 2009, which employed the same methodology as outlined above. In each case the question wording may vary slightly to take into account events in the interim, e.g. Senate vote outcome in March, but have been designed to measure the same sentiment. These questions are listed under each chart.

Summary Findings

Chart 1: Effectiveness of RTD Tax Increase in Addressing Binge-Drinking amongst Young People – Trend

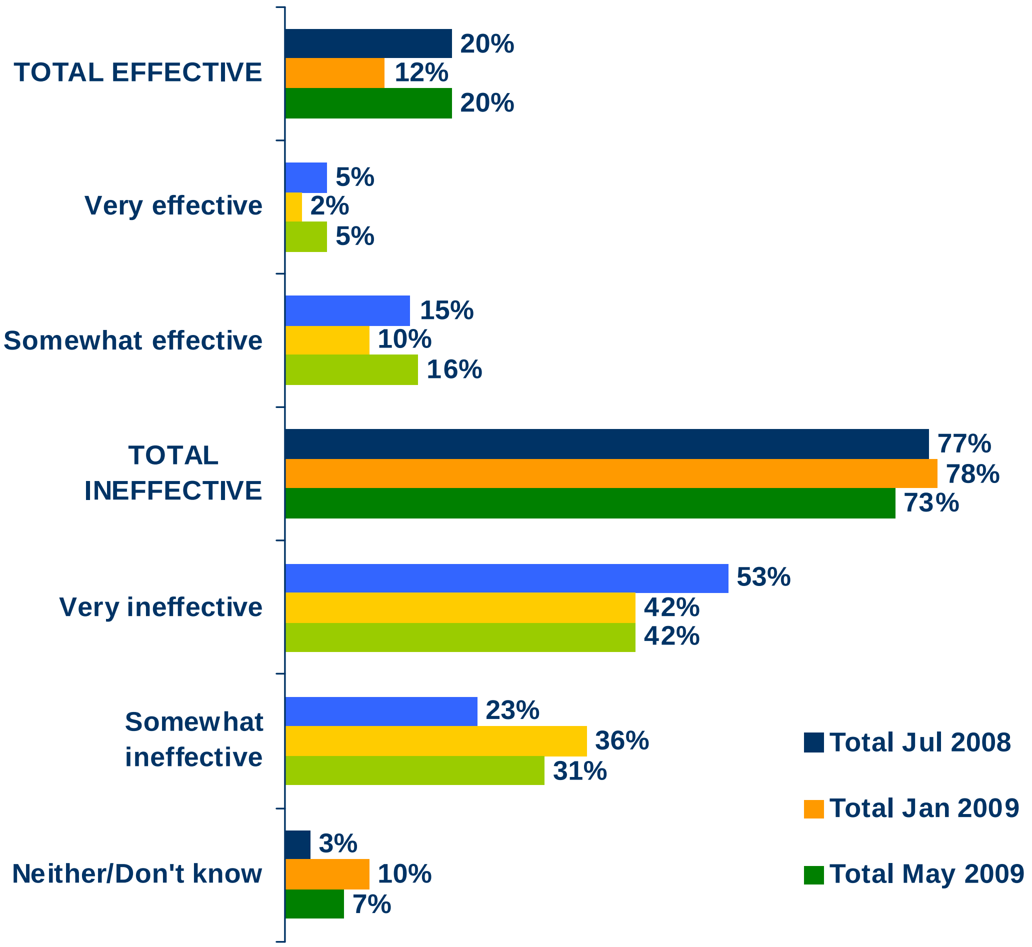

July 2008, Q1) Thinking now about some recent issues. To address binge-drinking amongst young people the Federal Government has increased the tax on Ready to Drink alcoholic drinks or so-called 'alcopops' by 70%. Do you think this is an effective or ineffective measure to deal with binge-drinking amongst young people? Is that very effective/ineffective or somewhat effective/ineffective?

January 2009, Q1) Thinking now about some recent issues. To address binge-drinking amongst young people the Federal Government has increased the tax on Ready to Drink alcoholic drinks or so-called 'alcopops' by 70%. This tax has now been in place for 9 months. From what you have seen, read or heard, do you think this has been an effective or ineffective measure to actually solve binge-drinking amongst young people?

May 2009, Q1) Thinking now about some recent issues. You may be aware that the Federal Government increased tax on Ready To Drink alcoholic drinks, or the so-called 'alcopops', by 70% last year. This tax bill was rejected by the Senate in March, but the Federal Government is seeking to re-introduce the tax to the Senate shortly, and will continue to collect the tax until the outcome is known. The Federal Government argues that the 'Alcopops Tax' is needed to address binge-drinking among young people. Do you think the tax has been an effective or ineffective measure to deal with binge-drinking among young people?

Chart 2: Agreement with Suggestion to Scrap RTD Tax in Favour of a Wider Strategy – Trend

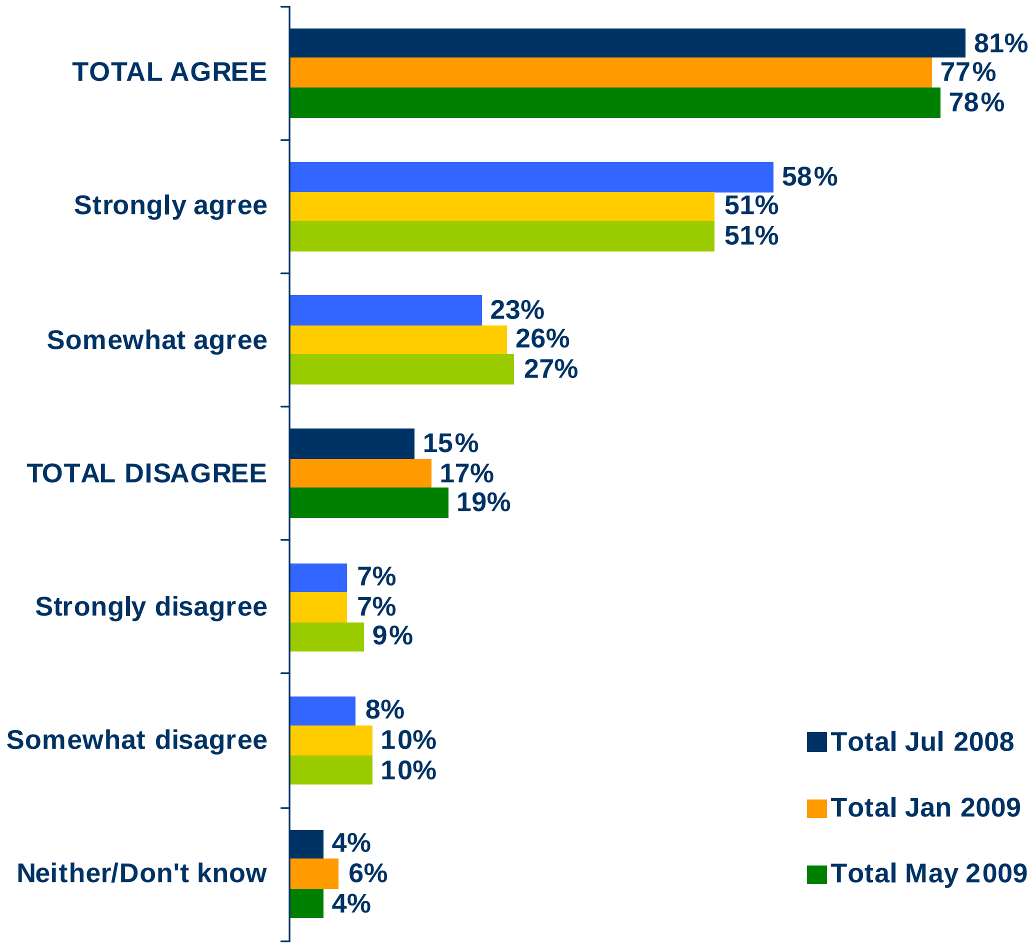

July 2008, Q2) Even though the Government will collect over $3 billion with this so-called 'alcopops tax', some people have suggested that it should be scrapped in favour of a wider and more comprehensive strategy to tackle binge drinking amongst young people. Do you agree or disagree with this suggestion to scrap the 'alcopops tax' in favour of a wider and more comprehensive strategy to tackle binge-drinking amongst young people? Is that strongly agree/disagree or somewhat agree/disagree?

January 2009, Q2) Even though the Government will collect approximately $1.7 billion with this so-called' alcopops' tax, some people have suggested that it should be scrapped or voted down by the Australian Senate in favour of a wider and more comprehensive strategy to tackle binge-drinking amongst young people. Do you agree or disagree with this suggestion that the Australian Senate should effectively scrap or vote down the 'alcopops tax' in favour of a wider and more comprehensive strategy to tackle binge drinking amongst young people?

May 2009, Q2) Even though the Government is to collect over $1.6 billion with this so-called 'AlcopopsTax', some people have suggested that it should be voted down again by the Senate in favour of a wider and more comprehensive strategy to tackle binge-drinking among young people. Do you agree or disagree with this suggestion to scrap the 'Alcopops Tax' in favour of a wider and more comprehensive strategy to tackle binge-drinking among young people?

Chart 3: Legitimacy of a Double Dissolution Election Based on Second Senate Rejection

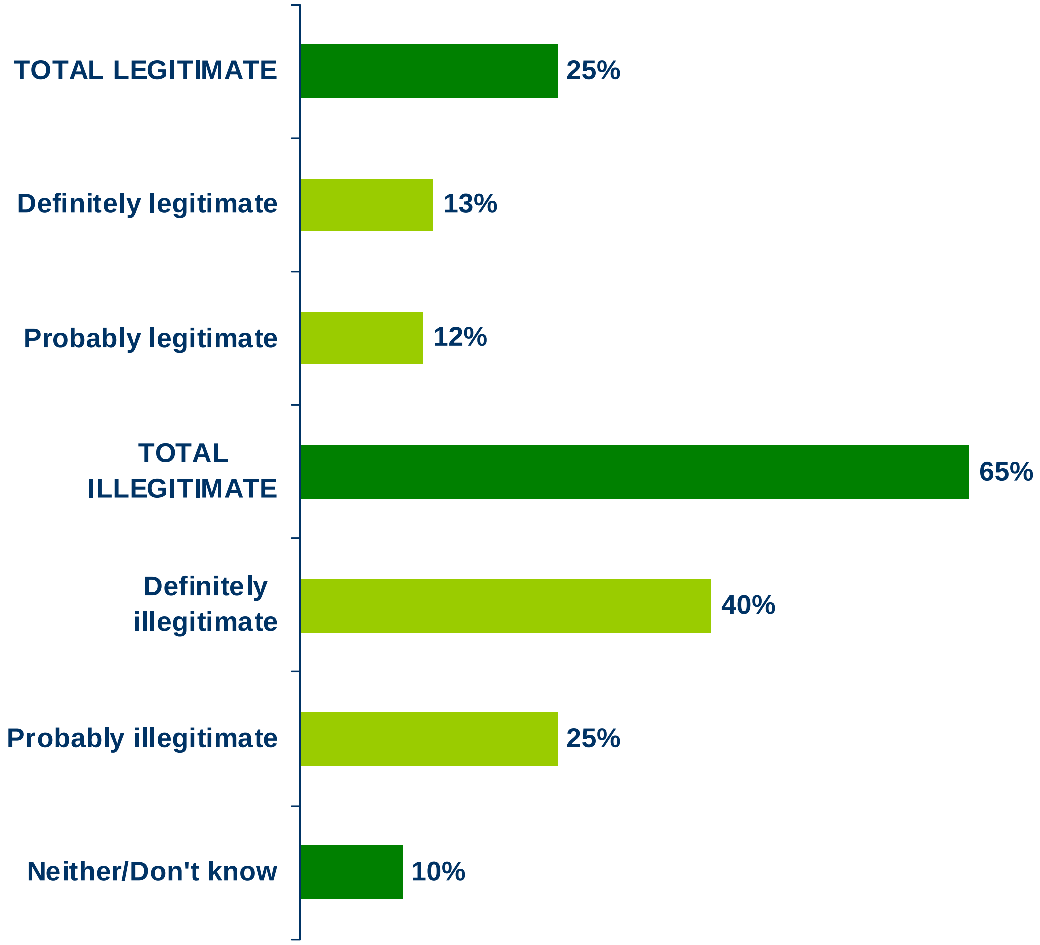

May 2009, Q3) You may be aware that if a re-introduced bill, such as this 'Alcopops Tax', fails to pass in the Senate for a second time, it can be used as a reason to hold a 'double dissolution' election. This means that the Federal Government can call an election for all seats in the House of Representatives this year rather than in late 2010, and all the seats in the Senate this year, rather than half the seats in 2010 and half in 2013. Given this, do you think that a second rejection of the 'Alcopops Tax' by the Senate is a legitimate or illegitimate reason for calling an early 'double dissolution' election?
